# The relationship between the grading stenosis based on morphology of the dural sac on MRI and the preoperative symptoms in patients with lumbar spinal canal stenosis

**Published:** 2012-11

**Authors:** Parisa Azimi

**Affiliations:** ^a^Department of Neurosurgery, Shahid Beheshti University of Medical Sciences, Tehran, Iran.

## Abstract

**Background:**

Grading stenosis based on morphology of the dural sac on MRI rather than surface measurements deﬁnes stenosis in various topics. To study the relations between the grading stenosis and the severity of clinical symptoms of spinal stenosis in patients diagnosed with lumbar spinal canal stenosis (LSCS).

**Methods:**

Patients diagnosed with lumbar spinal stenosis who were candidate for surgery entered into this cross sectional study. Grading stenosis based on morphology of the dural sac on magnetic resonance imaging (MRI) was determined. The severity of symptoms was evaluated based on the duration of symptoms, walking distance, visual analogue scale (VAS) of leg pain/numbness, the Neurogenic Claudication Outcome Score (NCOS) and the Japanese Orthopaedic Association (JOA) Score. We studied distribution of grades, relation between morphologic grading and severity of symptoms. The severity of symptoms and morphologic grading were compared between patients with different grades stenosis.

**Results:**

Eighty four patients were eligible to enter the study during the two years course of study. Patients were aged 61.2 ± 11.3 (18 to 82 years). All of patients were grades C and D stenosis 56 and 28 respectively. The symptoms were significantly worse in patients with a morphologic grade D stenosis (p less than 0.001). The walking distance in the patients with grade D stenosis was significantly shorter than those with grade C stenosis (P less than 0.001). In addition, the VAS of leg numbness in patients with grade D stenosis was significantly higher than those with a morphologic grade C stenosis (P less than 0.001). The JOA and the NCOS scores were significantly lower in patients with grade D stenosis (P less than 0.001). No significant difference was observed in the characteristics, duration of symptoms or VAS of leg pain between the groups.

**Conclusions:**

The findings suggest that the effect of grade D stenosis on patients’ symptoms, walking ability and functionality are more profound than grade C stenosis.

**Keywords:**

Lumbar spinal canal stenosis, Grading stenosis relationship, Severity of clinical symptoms


					Volume 4, Suppl. 1 Nov 2012
Publisher: Kermanshah University of Medical Sciences
URL: http://www.jivresearch.org
Copyright:
Congress of Iranian Neurosurgeons, 14-16 November 2012, Kermanshah, Iran
Paper No. 52
The relationship between the grading stenosis based on
morphology of the dural sac on MRI and the preoperative
symptoms in patients with lumbar spinal canal stenosis
Parisa Azimi a,*
a
Department of Neurosurgery, Shahid Beheshti University of Medical Sciences, Tehran, Iran.
Abstract:
Background: Grading stenosis based on morphology of the dural sac on MRI rather than surface measurements
defines stenosis in various topics. To study the relations between the grading stenosis and the severity of clinical
symptoms of spinal stenosis in patients diagnosed with lumbar spinal canal stenosis (LSCS).
Methods: Patients diagnosed with lumbar spinal stenosis who were candidate for surgery entered into this cross
sectional study. Grading stenosis based on morphology of the dural sac on magnetic resonance imaging (MRI) was
determined. The severity of symptoms was evaluated based on the duration of symptoms, walking distance, visual
analogue scale (VAS) of leg pain/numbness, the Neurogenic Claudication Outcome Score (NCOS) and the Japanese
Orthopaedic Association (JOA) Score. We studied distribution of grades, relation between morphologic grading and
severity of symptoms. The severity of symptoms and morphologic grading were compared between patients with
different grades stenosis.
Results: Eighty four patients were eligible to enter the study during the two years course of study. Patients were
aged 61.2  11.3 (18 to 82 years). All of patients were grades C and D stenosis 56 and 28 respectively. The
symptoms were significantly worse in patients with a morphologic grade D stenosis (p less than 0.001). The walking
distance in the patients with grade D stenosis was significantly shorter than those with grade C stenosis (P less than
0.001). In addition, the VAS of leg numbness in patients with grade D stenosis was significantly higher than those with
a morphologic grade C stenosis (P less than 0.001). The JOA and the NCOS scores were significantly lower in
patients with grade D stenosis (P less than 0.001). No significant difference was observed in the characteristics,
duration of symptoms or VAS of leg pain between the groups.
Conclusions: The findings suggest that the effect of grade D stenosis on patients' symptoms, walking ability and
functionality are more profound than grade C stenosis.
Keyword:
Lumbar spinal canal stenosis, Grading stenosis relationship, Severity of clinical symptoms
* Corresponding Author at:
Parisa Azimi: Department of Neurosurgery, Imam-Hossain Hospital, Shahid-Beheshti University of Medical Sciences, Imam-Hossain sq., Tehran, Iran.
Email: Parisa.azimi@gmail.com, (Azimi P.).

				

